# THEORETICAL IMPLICATIONS AND IMPACT OF SELF-COMPASSION IN CAREGIVERS OF PEOPLE LIVING WITH DEMENTIA

**DOI:** 10.1093/geroni/igac059.2101

**Published:** 2022-12-20

**Authors:** Claire Grant, Katherine Judge

**Affiliations:** Cleveland State University, Cleveland Heights, Ohio, United States; Cleveland State University, Cleveland, Ohio, United States

## Abstract

Caregivers of people living with dementia often report greater symptoms of depression, anxiety, and burden. Guided by the Stress Process Model, the study examined the impact of self-compassion on these key psychosocial well-being outcomes in a sample of dementia caregivers (N=99). Participants were 66.7% female, 67.7% White, and 74% were children/in law or grandchildren/in law, with a mean age of 38.61. Findings indicated self-compassion was a significant and unique predictor of symptoms of depression (β = -.25, p = .03), anxiety (β = -.36, p = .01), and burden (β = -.25, p = .02). Additional analyses found support for dysfunctional coping meditating the relationship between self-compassion and caregiver burden ((B = -.20, SE = .06, 95%CI[-.3242, -.0924] dysfunctional coping mediating the relationship between self-compassion and anxiety (B = -.16, SE = .05, 95%CI[-.2257, -.0302] and emotion-focused coping meditating the relationship between self-compassion and depression (B = -.06, SE = .03, 95%CI[-.1388, -.0045]. Results demonstrate the important role of self-compassion along with the impact of coping style on psychosocial well-being outcomes. Discussion will highlight the theoretical implications of these findings along with how these results can be used to develop efficacious interventions for caregivers of persons with dementia. Specifically, tailoring an intervention for dementia caregivers grounded in self-compassion theory as well as Compassion Focused Therapy and Mindful Self-Compassion Therapy may assist caregivers in developing a self-compassionate mindset and skills to more effectively cope with caregiving challenges.

